# Amyotrophic lateral sclerosis (ALS)-associated VAPB-P56S inclusions represent an ER quality control compartment

**DOI:** 10.1186/2051-5960-1-24

**Published:** 2013-06-12

**Authors:** Marijn Kuijpers, Vera van Dis, Elize D Haasdijk, Martin Harterink, Karin Vocking, Jan A Post, Wiep Scheper, Casper C Hoogenraad, Dick Jaarsma

**Affiliations:** 1 Department of Neuroscience, Erasmus Medical Center, Rotterdam, The Netherlands; 2 Division cell biology, Department Biology, Faculty of Science, Utrecht University, Utrecht, The Netherlands; 3 Biomolecular Imaging, Faculty of Science, Dept. Biology, Utrecht University, Utrecht, The Netherlands; 4 Department of Genome Analysis, Academic Medical Center, University of Amsterdam, Amsterdam, The Netherlands; 5 Department of Neurology, Academic Medical Center, University of Amsterdam, Amsterdam, The Netherlands

**Keywords:** Amyotrophic lateral sclerosis (ALS), Protein aggregation, ER associated degradation, Motor neuron disease, Mouse model

## Abstract

**Background:**

Protein aggregation and the formation of intracellular inclusions are a central feature of many neurodegenerative disorders, but precise knowledge about their pathogenic role is lacking in most instances. Here we have characterized inclusions formed in transgenic mice carrying the P56S mutant form of VAPB that causes various motor neuron syndromes including ALS8.

**Results:**

Inclusions in motor neurons of VAPB-P56S transgenic mice are characterized by the presence of smooth ER-like tubular profiles, and are immunoreactive for factors that operate in the ER associated degradation (ERAD) pathway, including p97/VCP, Derlin-1, and the ER membrane chaperone BAP31. The presence of these inclusions does not correlate with signs of axonal and neuronal degeneration, and axotomy leads to their gradual disappearance, indicating that they represent reversible structures. Inhibition of the proteasome and knockdown of the ER membrane chaperone BAP31 increased the size of mutant VAPB inclusions in primary neuron cultures, while knockdown of TEB4, an ERAD ubiquitin-protein ligase, reduced their size. Mutant VAPB did not codistribute with mutant forms of seipin that are associated with an autosomal dominant motor neuron disease, and accumulate in a protective ER derived compartment termed ERPO (ER protective organelle) in neurons.

**Conclusions:**

The data indicate that the VAPB-P56S inclusions represent a novel reversible ER quality control compartment that is formed when the amount of mutant VAPB exceeds the capacity of the ERAD pathway and that isolates misfolded and aggregated VAPB from the rest of the ER. The presence of this quality control compartment reveals an additional level of flexibility of neurons to cope with misfolded protein stress in the ER.

## Background

Protein aggregation is a central feature of many neurodegenerative disorders, including Alzheimer's disease, Parkinson’s disease and amyotrophic lateral sclerosis (ALS). Aggregation-prone proteins may accumulate into discrete micrometer-scale structures that are termed inclusions, inclusion bodies, aggregates or have disease or morphology specific names (e.g. Lewy bodies, Pick bodies, neurofibrillary tangles), and can be correlated to specific disorders [1]. Not only the protein composition, but also the morphologies as well as (sub) cellular and regional distributions of inclusions can be correlated to specific disorders and subtypes of disorders [1-3]. Depending on the type of disorder and inclusion, inclusions may be either neuroprotective, neutral or detrimental structures, and precise knowledge about their characteristics is instrumental for our understanding of neurodegenerative disorders [1,4].

A peculiar inclusion that is ultrastructurally characterized by the presence of ER-derived membranous profiles occurs in cellular and invertebrate models of a familial ALS-like disorder designated ALS8 [5-7]. ALS8 is caused by mutation in VAPB [8], a small tail-anchored ER membrane protein that is member of a conserved VAP (VAMP/synaptobrevin-associated proteins) family of proteins. Several *VAPB* mutations have been identified, but so far only a P56S mutation is yet known to co-segregate with disease [8,9]. VAP proteins are characterized by an N-terminal MSP (major sperm protein) domain, a coiled-coil motif, and a C-terminal transmembrane region, and in mammals consists of two genes, *VAPA* and *VAPB*[10,11]. The MSP domain (named after C. elegans MSP) contains a binding site for the FFAT (diphenylalanine [FF] in an acidic tract) that are present in a variety of proteins [12,13]. In addition, the MSP domain may function as a secreted ligand after cleavage from the transmembrane domain [7]. VAPs have been implicated in multiple function including non-vesicular transfer of lipids and membrane trafficking, ER-organelle and ER-cytoskeleton interaction and homeostatic and signaling functions at the neuromuscular synapse [10,14-16].

The P56S mutation causes rapid oligomerization and aggregation of mutant VAPB, and typically accumulates in multiple dot-like inclusions in transfected cells and animal models, including transgenic mice [6,8,17,18]. Several mechanisms by which mutant VAPB causes ALS have been proposed, including a dominant negative mode of action by recruiting wild-type VAPB and VAPA or other factors into aggregates, a gained toxic activity, or partial loss of function [5,6,15,19-21]. The aim of this study was to further characterize mutant VAPB inclusions *in vivo* in neurons of P56S-mutant VAPB transgenic mice. The data indicate that mutant VAPB inclusions that occur in motor neurons of these mice represent a specialized ER associated protein quality control compartment that isolates misfolded and aggregated VAPB targeted for degradation from the rest of the ER. The presence of this quality control compartment in addition to the ER associated degradation machinery may explain the late onset of mutant VAPB-induced disease in man.

## Methods

### Transgenic mice

Animals were housed and handled in accordance with the “Principles of laboratory animal care” (NIH publication No. 86–23) and the guidelines approved by the Erasmus University animal care committee.

**Figure 1:**
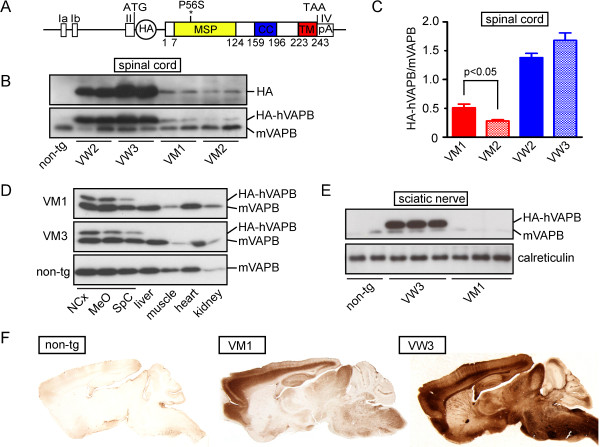
**Generation of VAPB-WT and VAPB-P56S transgenic mice. A**) To generate VAPB transgenic mice the cDNAs of wild-type or P56S-mutant human VAPB coupled to HA were cloned into the Thy1.2-expression casette. **B**-**E**) Western blots showing relative VAPB transgene expression levels in tissues of Thy1.2-hVAPB-WT (VW2, VW3) and Thy1.2-hVAPB-P56S (VM1, VM2, VM3) mice. Transgenic VAPB is detected with anti-HA antibody that specifically detects the transgene (**B**) or anti-VAPB antibody that interacts with both endogenous VAPB and transgenic VAPB running in a higher molecular weight band because of the HA-tag (**B**, **D**, **E**). Each lane is loaded with 2.5 μl S1 fraction derived from 250 μg tissue. **B**, **C**) Representative results (**B**) and quantification (**C**) of Western blot of spinal cord homogenates showing relatively high transgene expression levels in wild-type VAPB expressing lines (VW2 and VW3), and moderate transgene expression in mutant VAPB lines (VM1, VM2). Values in C are expressed as the ratio of the signals of endogenous and transgenic VAPB and represent means ± SE (n > 3). Spinal cords from VM2 mice show about half the level of transgene expression compared to VM1 spinal cord (Student *t*-test). **D**) Western blot of homogenates from different tissues from VM1 and VM3 mice showing that the transgene is specifically expressed in nervous system. **E**) Western blot of sciatic nerve homogenate of wild-type (line VW3) and mutant (line VM1) transgenic mice showing a high level of transgenic VAPB in wild-type VAPB sciatic nerve, and no transgenic VAPB in mutant VAPB sciatic nerve. **F**) Anti-HA immunohistochemistry showing widespread transgene expression in the brain of wild-type and mutant VAPB transgenic mice.

Transgenic VAPB mice were generated using the cDNAs of wild-type or P56S-mutant human *VAPB* cloned into the Thy1.2-expression module (Figure 1A). The VAPB-constructs also contained an HA-tag to enable easy visualization of transgenic VAPB by immunocytochemical approaches. Experiments in transfected cells have shown that the HA-tag does not alter the biochemical characteristics of wild-type and mutant VAPB [6]. Pronuclear injections yielded multiple founders carrying wild-type hVAPB or hVAPB-P56S. Data in this study were obtained from F1 - F10 offspring of 3 hVAPB-WT (VW1, VW2, VW3) and 4 hVAPB-P56S (VM1, VM2, VM3, VM5) founders. Lines were maintained in FVB background by crossing hemizygote males with non-transgenic females.

A selected group of different transgenic lines was allowed to age for 2 years (Additional file 1: Table S1). These mice were weighed and inspected for signs of muscle weakness once a week, using a set of simple tests: i.e. the mice were examined for their ability to extend their hindlimbs when suspended in the air by their tail, and their ability to hang upside down on a grid for 60 s [22]. In addition, at specific ages animals were subjected to an accelerating rotarod test as described [23]. The mice were killed when they developed motor problems or when they reached 2 years of age (Additional file: 1 Table S1). A subset of mice was excluded from the study because of non-motor related discomfort (e.g. eye infections or tumors; see Additional file 1: Table S1). Selected mice were analyzed for neuromuscular denervation and pathological abnormalities in the spinal cord (e.g. motor neuron loss, gliosis).

### Axotomy of the sciatic nerve

Six weeks old hemizygote hVAPB-P56S mice from the VM1 line and their non-transgenic littermates were anesthetized. The left sciatic nerve was exposed, bound with suture and cut at mid-thigh level. After various intervals mice were perfused transcardially with 4% paraformaldehyde and processed for immunocytochemistry.

### Antibodies

Primary antibodies reported in this study are: mouse anti-actin (Millipore); mouse anti-αB-crystallin (Stressgen Biotechnologies); rabbit anti-ATF3 (Santa Cruz Biotechnology); rabbit anti-BAP31 rabbit (gift from M. Tagaya; Tokyo University of Pharmacy and Life Sciences [24]); rabbit anti-BiP/GRP78 (Stressgen Biotechnologies); rabbit anti-calreticulin (Affinity BioReagents); mouse anti-CD8 (SantaCruz); goat-anti-choline acetyltransferase (ChAT, Millipore); rabbit-anti-CGRP (Calbiochem); rabbit anti-Derlin-1 (D4443, Sigma-Aldrich); rabbit anti-GFAP (DAKO); mouse-anti GM130 (BD Biosciences); mouse anti-HA (Covance); rat anti-HA (Roche); rabbit anti-HA (Santa Cruz Biotechnology); rabbit anti-Iba1 (WAKO Chemicals); rat anti-Mac2 (Cedarlane); rabbit anti KDEL (Stressgen Biotechnologies); rabbit anti-myc (Cell Signaling Technology); mouse anti-myc (Santa Cruz Biotechnology); mouse anti-NeuN (MAB377, Millipore); chicken anti-neurofilament M (Millipore), rabbit anti-NIR2 (Santa Cruz Biotechnology); rabbit anti-ORP9 (gift from Neale Ridgway, Dalhousie University, Canada); rabbit anti-ORP2, rabbit anti-ORP3, rabbit anti-ORP6 (gifts from Vesa Olkkonen, Institute for Molecular Medicine Finland); human anti-ribosomal protein P0 (Immunovision); Rabbit anti-phosphoS6 (Cell Signaling Technology), mouse anti-ubiquitin (FK2; 1:300; Biomol); rabbit anti-VAPB [6]; guinea pig anti-VAChT (Millipore); mouse anti-VCP (Ma3-004; Thermo Scientific).

Secondary antibodies: For avidin-biotin-peroxidase immunocytochemistry biotinylated secondary antibodies from Vector Laboratories diluted 1:200 were used. FITC-, Cy3-, and Cy5-conjugated secondary antibodies raised in donkey (Jackson Immunoresearch, USA), Alexa488, 568 or 633 conjugated antibodies raised in goat were used for immunofluorescence. For Western blots, HRP-conjugated goat-anti mouse or goat-anti rabbit IgG were used at 1:5000 (DAKO, 1:5000).

### Western blotting

Tissue samples were homogenized in ten volumes of PBS containing 0.5% Nonidet P-40 and 1× protease inhibitor cocktail (Complete, Roche), sonicated and centrifuged at 800 g for 15 min at 4°C to obtain the S1-supernatant. For the preparation of detergent-insoluble extracts, S1 supernatants were centrifuged at 15000 g for 20 min. After the collection of supernatants (S2), pellets (P2) were thoroughly washed five times with PBS-0.5% Nonidet P-40 and then resuspended in sample buffer for SDS–PAGE electrophoresis and western blotting. Protein concentrations in samoles were determined using the BCA method (Pierce, Rockford, IL).Samples containing 5–50 mg protein were electrophoresed on SDS–PAGE gels and blotted on PVDF membranes (Millipore). The membranes were blocked with 5% non-fat dry milk (Bio-Rad) in PBS with 0.05% Tween20 (PBST), incubated in primary antibody, diluted in PBST with 1% dry milk followed by incubation in secondary antibody. Blots were exposed to film after incubation in chemiluminescence’s reagent (ECL, Amersham), and films were analyzed with Metamorph software.

### RT-PCR of unfolded protein responsive genes

Levels of unfolded protein stress responsive mRNAs were analyzed by real time quantitative reverse transcription PCR (qRT-PCR) using the Roche LightCycler 480 and the Roche universal probe library as described [25]. RNA was isolated from cortex samples using Trizol reagent and used for cDNA synthesis. Primers for the qRT-PCR assay were: BiP FW: gccaactgtaacaatcaaggtct/RV: tgacttcaatctggggaactc (probe #15) and Chop FW: ccaccacacctgaaagcag/RV: tcctcataccaggcttcca (probe #33). Values are normalized to eEF2α mRNA FW: acacgtagattccggcaagt/RV: aggagccctttcccatctc (probe #31) for individual animals [25].

### Primary neuron cultures and transfection

Primary hippocampal cultures were prepared from embryonic day 18 (E18) rat brains [26]. Cells were plated on coverslips coated with poly-L- lysine (30 μg/ml) and laminin (2 μg/ml) at a density of 75,000/well. Hippocampal cultures were grown in Neurobasal medium (NB) supplemented with B27, 0.5 mM glutamine, 12.5 μM glutamate and penicillin/streptomycin. Hippocampal neurons were transfected using Lipofectamine 2000 (Invitrogen). The following mammalian expression plasmids have been described previously: HA- and myc-tagged VAPB-wt and VAPB-P56S constructs [6]; myc-tagged seipin-wt, seipin-N88S and seipin-S90L constructs [27]; and BAP31-mRFP construct [24]. ΔTM-VAPB-P56S-GFP was generated by a PCR-based strategy using HA-VAPB-P56S construct as a template and subcloned into a GFP-tagged pβactin expression vector. HA-VAPB-P56S-CD8TM was made by removing the transmembrane domain of VAPB-P56S and adding the transmembrane domain of CD8 with a PCR-based strategy using HA-VAPB-P56S and GFP-CD8 [28] as a template and subcloned into a pβactin expression vector. BAP31 (5’-gagaatgatcagctaaaga-3’) and TEB4 (5’-ttaagagcctcttgcctca-3’) shRNA construct sequences were designed based on previously published sequences [29,30]. The complementary oligonucleotides were annealed and inserted into a pSuper vector [31]. DNA (3.6 μg /well) was mixed with 3 μl of Lipofectamine 2000 in 200 μl of medium, incubated for 30 min, and then added to the neurons in NB at 37°C in 5% CO2 for 45 min. Next, neurons were washed with NB and transferred in the original medium at 37°C in 5% CO2. 2–4 days after transfection, neurons were fixed with 4% paraformaldehyde/4% sucrose in PBS, washed three times in PBS for 10 min and incubated with the indicated primary antibodies in GDB buffer (0.2% BSA, 0.8 M NaCl, 0.5% Triton X-100, 30 mM phosphate buffer, pH 7.4) overnight at 4°C. Following incubation with secondary antibody neurons were mounted using Vectashield mounting medium (Vector laboratories). Images for co-localization measurements were acquired using a Nikon microscope equipped with a 100x oil objectives. Confocal images were acquired using a LSM510 confocal microscope (Zeiss) with 40x or 63x oil objectives.

### Immunohistochemical and histopathological procedures

For immunocytochemistry and immunofluorescence mice were anaesthetized with pentobarbital and perfused transcardially with 4% paraformaldehyde. The lumbar and cervical spinal cord were carefully dissected out and post-fixed overnight in 4% paraformaldehyde. Routinely, spinal cord tissue was embedded in gelatin blocks, sectioned at 40 μm with a freezing microtome and sections were processed, free floating, employing a standard avidin-biotin-immunoperoxidase complex method (ABC, Vector Laboratories, USA) with diaminobenzidine (0.05%) as the chromogen, or single, double and triple-labelling immunofluorescence [22]. Immunoperoxidase-stained sections were analyzed and photographed using a Leica DM-RB microscope and a Leica DC300 digital camera. Sections stained for immunofluorescence were mounted on coverslips, placed on glass slides with Vectashield mounting medium, and were examined with Zeiss LSM 510 and LSM 700 confocal laser scanning microscopes.

For analysis of neuromuscular denervation medial gastrocnemius muscle from 4% paraformaldehyde fixed mice were dissected, embedded into gelatin blocks and sectioned at 80 μm with a freezing microtome [22]. Sections were immunolabeled, free floating, for guinea pig anti-VAChT and chicken-anti-NFM followed by Cy3 anti-goat and Cy5 anti-chicken or anti-rabbit secondary antibody, and motor endplates were labeled with FITC-bungarotoxin (1:500, Molecular Probes). For quantitative analyses, muscle sections were examined under a Leica DM-RB fluorescence microscope, end-plates being scored as ‘innervated’ in case of complete overlap between bungarotoxin and VAChT labeling, ‘partially denervated’ in case of partial overlap, and ‘denervated’ in case of the absence of VAChT labeling at the end-plate.

### Quantitative analysis of immunofluorescence images

Fluorescent intensities and inclusion sizes were determined using Metamorph image analysis software. Images were collected using Zeiss LSM 510 confocal laser scanning microscope with 63x Plan apo oil immersion objective. Analyses of inclusions in cultured neurons were performed on maximal projections of confocal stacks. For analysis of FK2-labeled motor neurons stacks of 1 μm thick sections were collected from the first 4 μm facing the coverslip, and the optical section 2 μm below the surface of the section was used for density measurements. Material from non-transgenic and transgenic mice always was imbedded in a single gelatin block to minimalize variability in staining intensity resulting from the sectioning and immunostaining procedure [22]. Per mouse, motor neurons from 3 randomly selected L4 sections (yielding 4–12 cells/sections) were measured.

### Analyses of sciatic nerves

Sciatic nerves were carefully dissected from perfused mice, post-fixed in 4% paraformaldehyde with 1% glutaraldehyde, extensively rinsed in 0.1 M PB, post-fixed in 1% osmium, dehydrated, embedded in Durcupan, sectioned transversely at 0.5 μm with an Ultratome, and stained with toluidine blue.

### Transmission electron microscopy

For electron microscopy mice were perfused transcardially with 4% paraformaldehyde with 0.2% (post-embedding immunogold electron microscopy) or 1% (standard transmission electron microscopy) glutaraldehyde. Specimens were sectioned with a Vibratome and further processed using standard methods as described before [22,32]. For standard transmission electron microscopy Vibratome section (60–100 μm) were post-fixed in 1% osmium, dehydrated and embedded in Durcupan. Ultrathin sections (50–70 nm) were contrasted with uranyl acetate and lead citrate, and analyzed in a Phillips CM100 electron microscope at 60 or 80 kV.

Post-embedding immunogold labeling was performed on 50–70 nm thick thin sections from 4% paraformaldefyde and 0.2% glutaraldehyde fixed brain and spinal cord sections as described before [22] using the rat-anti-HA antibody at 1:100.

For electron microscopic analysis of VAPB-P56S inclusions in HeLa cells, cells were transfected with Myc-hVAPB-P56S and the Addgene plasmid 40307 to enable selection of transfected cells under the electron microscope [33]. 24 h after transfection cells were fixed with 2% paraformaldehyde and 2.5% glutaraldehyde in cacodylate buffer, stained for DAB [33], post-fixed with 1% OsO4 (EMS) and 1.5% K_4_[Fe(CN)_6_] in cacodylate buffer (90 min on ice), followed by 1% low molecular weight tannic acid (30 min at RT) and with 1% OsO4 in distilled water (30 min on ice) as described [34], embedded in epon, sectioned at 50 nm, and contrasted with uranyl acetate [6].

### Statistical analyses

Statistical analyzes were performed with MS Excel or Graphpad Prism software (San Diego, USA). Means from different age groups, and different transgenic mouse lines were compared using Student’s t-test, or one-way ANOVA and Tukey’s post-test.

## Results

### Mutant VAPB inclusions are positive for luminal ER proteins and are surrounded by ribosome-rich areas

**Figure 2:**
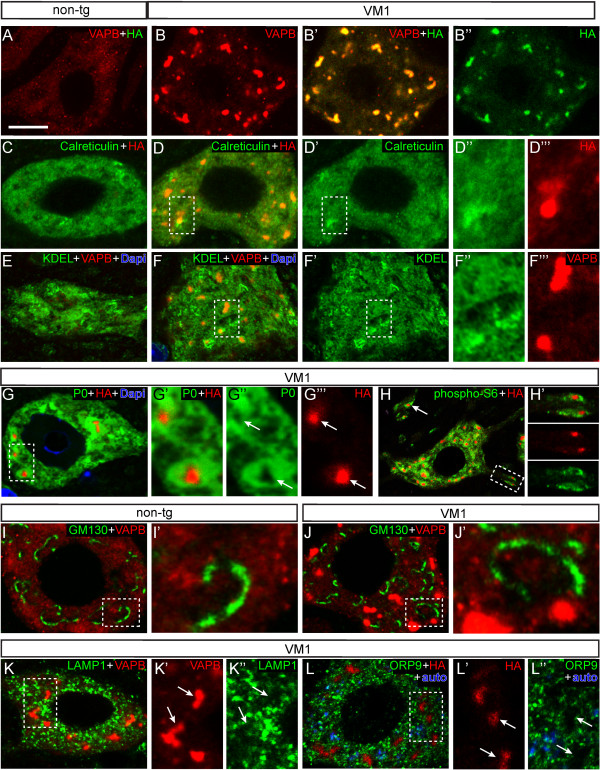
**VAPB Inclusions in VAPB-P56S transgenic motor neurons are immunoreactive for ER markers and surrounded by ribosomal markers. A**, **B**) Confocal immunofluorescence of HA (specific for transgenic VABP) and VAPB (labels endogenous and transgenic VAPB) in motor neurons of non-transgenic (**A**) and mutant VAPB (line VM1; **B**) mice showing multiple small intensely HA and VAPB-immunoreactive inclusions in VM1 motor neurons. Note, that VAPB-immunoreactivity in the rest of the cell body is the same as in non-transgenic mice. **C**-**F**) Confocal immunofluorescence of spinal motor neurons double-labeled with either anti-HA; **C**, **D**) or anti-VAPB; **E**, **F**) and antibodies against ER proteins, i.e. calreticulin (**C**, **D**) or KDEL-motif proteins (**E**, **F**); calreticulin and KDEL-immunoreactivities are diffusely distributed throughout the perykarya of both non-transgenic (**C**, **E**) and mutant VAPB (**D’**, **F’**) motor neurons irrespective of the presence of VAPB inclusions, but **G**, **H**) Double labeling for HA and the ribosomal proteins P0 (**G**) or phosphorylated-S6 (**H**) shows that mutant VAPB inclusions are P0 and phospho-S6-immunonegative but the surrounding cytoplasm is always intensely P0 (arrows in **G**) and phospho S6-positive (**H**). Arrow and insert in H show that also dendritic VAPB inclusions are surrounded by high levels of ribosomes. **I-L**) Double labeling of VAPB or HA, with antibodies against the cis-Golgi protein GM130 (**I**, **J**), lysosomal protein LAMP1 (**K**), and the FFAT-motif protein ORP9 (**L**), shows that VAPB inclusions (arrows in **K** and **L**) are immunonegative for these proteins. Note the presence of autofluorescent structures, representing lipofuscin (aging pigment) in the motor neuron shown in L, which is from a 70 week old VM1 mouse. Bar in **A**, 10 μm.

To examine mutant VAPB inclusions in neurons *in vivo* we generated transgenic mice carrying a construct of human *VAPB* cDNA with or without the P56S mutation cloned into the Thy1.2 expression cassette (Figure 1A) that drives transgene expression in neurons throughout the CNS, including spinal motor neurons [22,35]. The transgenes incorporated an HA-tag at the N-terminus to enable the efficient localization of transgenic protein at the light and ultrastructural level [36]. Four lines of hVAPB-P56S (VM1, VM2, VM3, VM5) and 3 lines of wild-type (wt)-hVAPB (VW1, VW2, VW3) transgenic mice were obtained (Figure 1; Additional file 1: Figure S1). Consistent with previous studies with transfected cells [6,8] and transgenic mice [17,18], hVAPB-P56S expressing trangenic mice developed VAPB inclusions in motor neurons as well as other populations of neurons, including spinal interneurons, neurons in brain stem reticular formation and the cerebellar nuclei, and pyramidal neurons in neocortex. Mutant VAPB inclusions consisted of small spherical and ellipsoid structures that were intensely HA and VAPB-immunoreactive, and were distributed throughout the cell body and proximal dendrites (Figure 2). Significantly, the mutant VAPB inclusions did not occur in mutant VAPB transgenic mice from line VM2, which showed lower transgene expression levels than the other lines (Figure 1; Additional file 1: Figure S2). Inclusions also were absent in wild-type VAPB transgenic mice (see below).

To determine whether the presence of inclusions was associated with altered solubility of mutant VAPB we performed Western blot analysis of non-ionic detergent (Nonidet P40)-insoluble (P2) fraction of spinal cord homogenate. In accordance with reduced solubility a large proportion of transgenic mutant VAPB accumulated in the insoluble fraction (Additional file 1: Figure S2). Endogenous murine VAPB was not detectable in this fraction, suggesting that it does not coaggregate with transgenic mutant VAPB (Additional file 1: Figure S2).

To characterize the mutant VAPB inclusions we first double stained for HA or VAPB and a variety of cellular markers. Double labeling with antibodies against calreticulin (a luminal ER sugar-binding protein) and KDEL (a C-terminal tetrapeptide motif shared by several ER chaperones), showed that the mutant VAPB inclusions were immunoreactive for these ER markers (Figure 2C-F). However calreticulin and KDEL staining was not enriched in the inclusions; the same staining intensity is observed in the inclusions as compared to the surrounding area. Accordingly, the calreticulin and KDEL staining in motor neurons with inclusions was indistinguishable from that in non-transgenic motor neurons (e.g. compare Figure 2C and 2E with 2D’ and F’, respectively). Double labeling with antibodies against ribosomal protein P0 and phosphorylated ribosomal protein S6 showed that while the inclusions were immunonegative for P0 and phospho-S6, the area around the inclusions contains a high density of ribosomes (Figure 2G, H). The specific association of mutant VAPB inclusions within ribosome-rich areas was particularly clear in motor neuronal dendrites, which showed areas of intense phospho-S6 staining. Analysis of a large number of dendritic VAPB inclusions (> 100) indicated that in all occasions they were present within an area of intense phospho-S6 staining (Figure 2H’).

Mutant VAPB did not codistribute with the Golgi apparatus marker GM130, and the presence of inclusions did not have a detectable effect on the Golgi apparatus morphology (Figure 2I, J). Also markers for lysosomes (LAMP1, Figure 2K) and endosomes (EAA1, not shown) did not codistribute with inclusions and showed unaltered distributions in motor neurons with inclusions. Finally we screened antibodies against a variety of FFAT-motif containing proteins, representing a major class of VAPB interacting proteins [6,37,38] to determine whether these proteins accumulate in the mutant VAPB inclusions. Antibodies against NIR2 and ORP9 produced consistent labeling in motor neurons. However, ORP9 (Figure 2L) nor NIR2 (data not shown) immunoreactivity was present in the mutant VAPB inclusions, consistent with the observation that the P56S mutation disrupts the FFAT-motif binding domain of VAPB [6].

### VAPB inclusions ultrastructurally are characterized by smooth ER-like profiles and electron dense material

**Figure 3:**
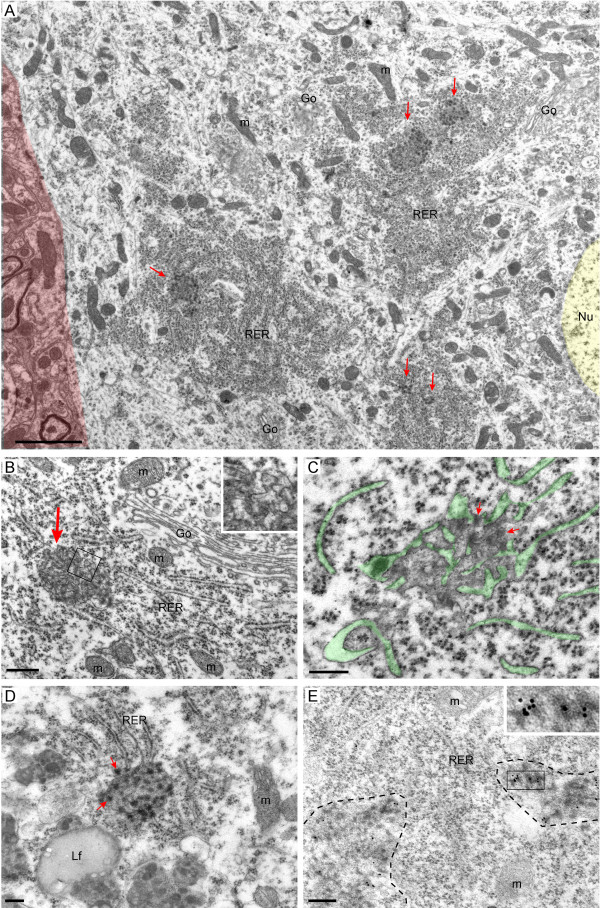
**Mutant VAPB inclusions are localized in the RER and consist of smooth ER-like tubular profiles and electron dense material. A**-**D**) Transmission electron photomicrographs of mutant VAPB inclusions in spinal motor neurons of a 6 weeks old VM1 (**A**, **B**), a 6 week old VM3 (**C**), and a 70 weeks old VM1 x VM5 mouse (**D**). Note in A that the inclusions (red arrows) are localized within normal appearing rough ER (RER). Other organelles such as Golgi apparatus (Go) and mitochondria (m) are unaltered (**A**, **B**). In C the ER lumen is accentuated in green to outline the continuity of tubular smooth ER like profiles in the inclusions with the surrounding rough ER cisterns. Note in C and D that the electron dense material in the inclusions has a patchy appearance (red arrows). **E**) Post-embedding immunogold electron microscopy with anti-HA antibody showing that HA-labeling is preferentially associated with the electron dense material in the inclusions (see insert). The dashed lines outline two distinct inclusions. Color overlay in **A**: red, area outside the motor neuron; yellow, nucleus; green, lumen of ER. Scale bars: 2 μm (**A**), 500 nm (**B**), 200 nm (**C**, **D**, **E**).

Transmission electron microscopy of spinal motor neurons of VAPB-P56S transgenic mice from the lines VM1, VM3 and VM5 revealed the ultrastructural correlate of the mutant VAPB inclusions: i.e. regions, 0.3 to 2 μm in diameter, within the rough ER (RER) that contained smooth ER-like tubular and vesicular profiles and electron dense material (Figure 3). These abnormal regions were contacted by surrounding rough ER profiles (Figure 3A-D). In multiple occasions the rough ER was continuous with tubular profiles in the inclusions (Figure 3C). The electron dense material in the inclusions was at the cytoplasmic side of the tubular profiles and usually showed small diffuse clusters, 50–75 μm in diameter, of higher electron density (Figure 3C, D). The rough ER surrounding the regions with abnormal ER showed a normal appearance, and no abnormalities were observed in other organelles. Analysis of spinal interneurons, revealed the same ER abnormalities, indicating this abnormality does not depend on specific features of the ER in motor neurons (not shown). Analysis of mutant VAPB transgenic mice of different age indicated that the inclusions were the same in old (70–90 weeks; Figure 3D) versus young (4–15 weeks) mice.

Several findings indicate that the structures consisting tubular smooth ER-like profiles and electron dense material indeed represent the ultrastructural correlate of inclusions identified light-microscopically: First, their size, frequency, and exclusive distribution within regions of rough ER are consistent with those of inclusions identified light-microscopically. Second, this ER abnormality does not occur in motor neurons of transgenic mutant VAPB mice of the VM2 line, which do not show light-microscopic inclusions (Table 1). Third, post-embedding immunogold electron microscopy with anti-HA antibody, showed that HA-labeling was strongly enriched within these abnormal ER compartments (Figure 3E; 78.40 ± 16.98 particles/μm^2^ [10 inclusions] versus 0.32 ± 0.14 gold particles/μm^2^ in the surrounding cytoplasm and 0.24 ± 0.08 particles/μm^2^ in non-transgenic motor neuron, n = 10 cells).

**Table 1:** Summary of ER abnormalities in Thy1.2-hVAPB transgenic lines

**Mouse line**	**HA-staining**	**ER abnormalities**		
		**Tubular ER**	**Stacked ER**	**Electron dense stacked ER**
**hVAPB-wt**				
**VW1**	+++++	-	+	+
**VW2**	++++	-	+	-
**VW3**	++++	-	+	-
**hVAPB-P56S**				
**VM1**	++	+	-	-
**VM2**	+	-	-	-
**VM3**	++	+	-	-
**VM5**	++	+	-	-
**VM1 + VM5**	+++	+	-	-

### Stacked ER cisterns in wt-hVAPB motor neurons

**Figure 4:**
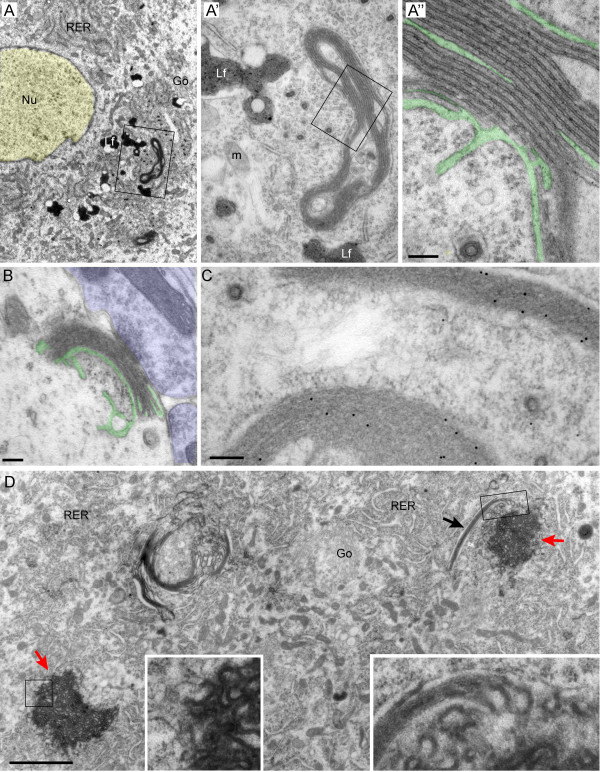
**Stacked ER in motor neurons of wild-type VAPB overexpressing mice. A**, **B**) Transmission electron photomicrographs showing stacked ER in motor neurons from a 30 week old wild-type VAPB overexpressing mouse (line VW3). The ER cisterns in the stacks are thin, while the cytosolic faces of the cisternal membranes are separated by a 17.2 ± 1.6 nm thick layers (**A**). In A“ the ER lumen is accentuated in green to outline the continuity of lumen of stacked cisterns with the surrounding ER cisterns. B illustrates stacked ER next to a synapse. **C**) Post-embedding anti-HA immunogold electron photomicrographs, showing that transgenic wild-type VAPB is concentrated in the stacked ER. **D**) Transmission electron photomicrograph of a motor neuron of an 8 week old mouse from line VW1 showing stacked ER (black arrow) and a variant of stacked ER (red arrows), consisting of curved more irregular ER cisterns, and electron dense intra-cisternal spaces (inserts in **D**). Color overlay: yellow, nucleus (**A**); purple, presynaptic bouton (**B**); green, lumen of ER (**A**, **B**). Scale bars: 2 μm (**D**), 500 nm (**B**), 100 nm (**A”**, **C**).

Motor neurons of wt-hVAPB mice did not show the ER abnormalities observed in VAPB-P56S transgenic mice, indicating that these inclusions are a specific consequence of mutant VAPB (Table 1). Instead a subset of motor neurons in wild-type VAPB transgenic mice, showed another ER abnormality, i.e. stacked ER, consisting of flat or circular arrays of parallel narrow cisterns (Figure 4A). The cisterns were separated by a 17 ± 2 nm thick layer (Figure 3A). These stacked ER resembled previously reported stacked ER (also termed organized smooth ER [OSER], or crystaloid ER) observed in cells expressing high levels of certain ER membrane proteins [39,40] including cells coexpressing VAPB together with FFAT-motif protein [41,42]. Stacked ER was usually localized in the rough ER, the superficial cisterns being continuous with rough ER cisterns (Figure 4A). Occasionally, however, stacked ER was found in other cellular compartments such as near synapses (Figure 4B). Immunogold labeling showed increased anti-HA immunoreactivity within stacks (Figure 4C). Remarkably, in one line of wt-hVAPB mice (line VW1) we noted a variant of stacked ER, where ER cisterns were curved, the lumen of the cisternae was expanded and the space linking cisterns together was considerably more electron dense (Figure 4D). This electron dense form of stacked ER was continuous with both ‘regular’ ER stacks and rough ER profiles (inserts in Figure 4D). Both the ‘regular’ and the electron-dense irregular variant of stacked ER were never observed in VAPB-P56S transgenic mice.

### Gradual loss of mutant VAPB inclusions in axotomized motor neurons

**Figure 5:**
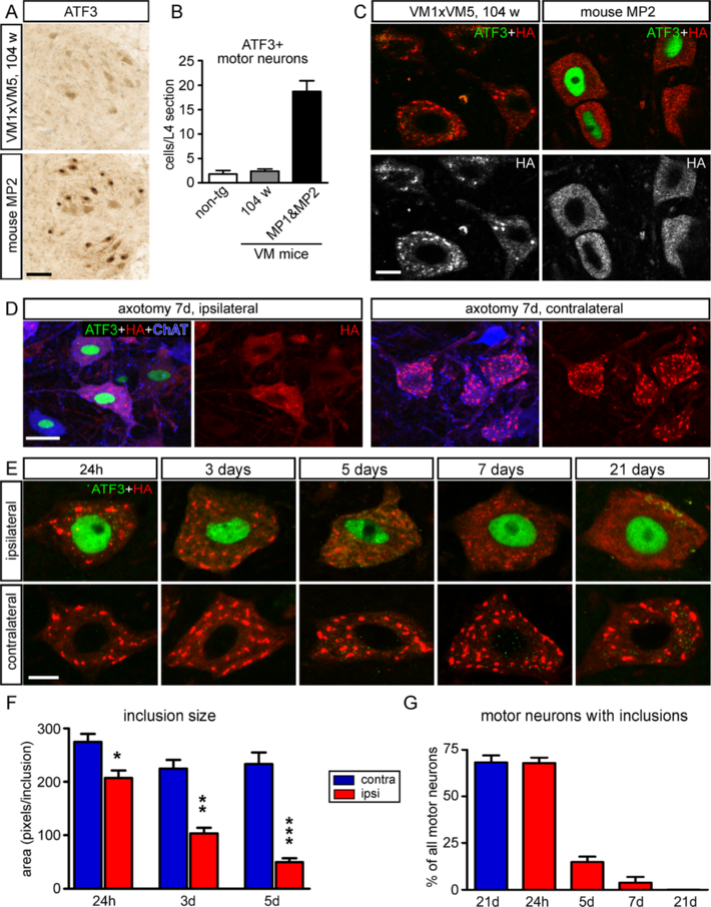
**Gradual loss of mutant VAPB inclusions in axotomized motor neurons. A**-**C**) Representative images (**A**) and bar graph (**B**) showing ATF3 expression in a large number of motor neurons of two mutant VAPB mice that developed progressive motor abnormalities. The mice with a motor phenotype consisted of a mouse from line VM3 (mouse MP1, onset 61 weeks) and line VM1xVM5 (mouse MP2, onset 74 weeks). Non-trangenic (non-tg, n = 4) and transgenic littermates (n = 5) killed at the age of 104 weeks show ATF3 expression in a low proportion of motor neurons. **C**) Confocal images of motor neurons double-labeled for HA and ATF3 showing the absence of VAPB inclusions in ATF3 expressing motor neurons of mouse MP2. **D**-**G**) Gradual loss of mutant VAPB inclusions in axotomized motor neurons. **D**) Confocal image of lumbar motor neurons of a VM1 mutant VAPB mouse, 7 days after sciatic nerve transection, showing that axotomized motor neurons identified by ATF3 expression (ipsilateral) have no or very small VAPB inclusions as compared to control (contralateral) VM1 motor neurons. **E**) Representative images of axotomized motor neurons (ipsilateral, ATF3 positive) from VM1 mice killed at different time points after axotomy. Note the gradual reduction of the size of VAPB inclusion following post-axotomy, ultimately leading to diffuse perikaryal HA staining 2–3 weeks post-axotomy. **F**, **G**) Bar graphs showing the inclusion size expressed as mean number of pixels per inclusion (**F**), and the percentage of HA-labeled motor neurons with inclusions (**G**). Values in **F** represent means ± SE from more than 20 motor neurons from 2–3 mice. *, **, ***: P < 0.05, P < 0.01, P < 0.001 compared to contralateral (unpaired Student’s t-test). Scale bars: 100 μm, (**A**), 25 μm (**D**), 10 μm (**C**, **E**).

Together our confocal and electron microscopic data indicate that inclusions in VAPB-P56S transgenic mice represent unique ER associated structures. Analysis of the expression of two unfolded protein response target genes, i.e. the ER chaperone Bip (Grp78, Hspa5) and Chop (Gadd153) by immunofluorescence and qRT-PCR showed unaltered expression in both wild-type and mutant VAPB transgenic mice (Additional file 1: Figure S3), indicating that the expression of mutant VAPB per se or the presence of mutant VAPB inclusions do not triggers induction of unfolded protein response. Analysis of motor behavior by accelerating rotarod, hanging wire and hind limb extension tests in a cohort of 46 mutant VAPB transgenic mice from different lines including double transgenic mice generated by intercrossing mice from VM1 and VM5, showed that the far majority (44 of 46) of mice reached the age of 2 years without developing obvious motor deficits and signs of neuronal degeneration (Additional file 1: Figure S4; and Table S1). These findings are consistent with data from previous studies reporting normal survival and the absence of motor abnormalities in mutant VAPB transgenic mice [17,18]. Remarkably, however, two (of 46) mice developed late onset progressive motor impairment, and axonal degeneration in peripheral nerve (Additional file 1: Figure S5; and Table S1). Although the number of these two mice with a motor phenotype is too low to conclusively link the phenotype to VAPB-P56S expression, the mice shared two interesting features, i.e. the expression of ATF3, a stress transcription factor that is expressed in motor neurons following multiple pathological conditions [43-45], in a large number of motor neurons (Figure 5A, B). More importantly, double labeling with anti-HA antibody showed that none of the ATF3 expressing motor neurons in the mice with a motor phenotype showed VAPB inclusions (Figure 5C). These data suggest a correlation between ATF3 expression, axonal degeneration and the absence of VAPB inclusions in motor neurons of mutant VAPB transgenic mice.

To further investigate the connection between ATF3 expression, axonal damage and the absence of VAPB inclusions, we examined the effect of axotomy on inclusions in sciatic nerve motor neurons of VM1 mice. Axotomy results in a strong induction of ATF3 expression in motor neurons within 12 hours, which lasts for more than 5 weeks post transection [44,46]. Analysis of inclusions in axotomized ATF3-positive motor neurons revealed a gradual reduction of the size of inclusions starting within 24 hours post-axotomy (Figure 5D-G). Ultimately, axotomy resulted in the total absence of inclusions and diffuse HA labeling 2–3 weeks post-axotomy (Figure 5D-G). The diffuse HA labeling in 2–3 weeks axotomized VM1 motor neurons strongly resembled HA-labeling in motor neurons of MP1 and MP2 mice described above. Importantly, the level of HA-labeling in 2–3 weeks axotomized VM1 motor neurons is considerable higher than in VM2 motor neurons, indicating that the absence of inclusions cannot simply be explained by reduced VAPB-P56S expression levels. Together the data indicate that mutant VAPB inclusions in motor neurons of VAPB-P56S transgenic mice are reversible ER-associated structures.

### VAPB inclusions are immunoreactive for proteins of the ER associated degradation (ERAD) quality control pathway

**Figure 6:**
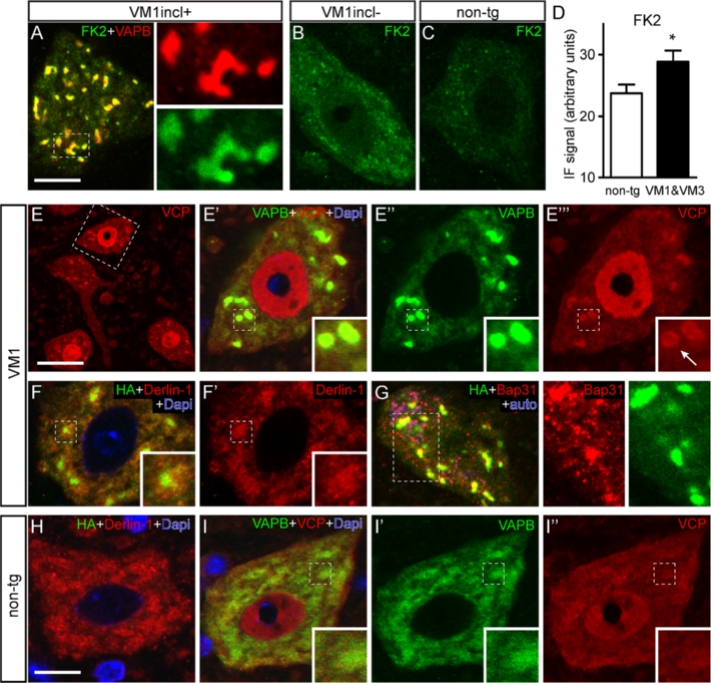
**Mutant VAPB inclusions are ubiquitin-positive and enriched in ERAD components. A**-**C**) Representative confocal images of the distribution of ubiquitinated epitopes labeled with the FK2 antibody in motor neurons of mutant VAPB line VM1 with (**A**) or without (**B**) inclusions, and non-transgenic littermates (**C**). Note high level of FK2-immunoreactivity in inclusions (**A**) and diffuse increase as compared to non-transgenic motor neurons in VM1 motor neurons without inclusions (**B**). **D**) Bar graph of FK2-immunofluoresencesignal in non-transgenic versus VM1 and VM3 motor neurons without inclusions. Values are means ± SE and are from lumbar L4 segments from 4 non-transgenic, 2 VM1 and 2 VM3 mice embedded in a single gelatin block (see methods). **E**-**I**) Confocal image of mutant VAPB (line VM1, **E**-**G**) or non-transgenic (**H, I**) spinal motor neurons double-labeled for VAPB or HA and ERAD components, i.e. VCP/p97 (**E**, **I**), derlin-1 (**F**, **H**), and BAP31 (**G**). Note, enrichment of VCP (**E**) and BAP31 (**G**) staining in the mutant VAPB inclusions; derlin-1 although present does not show preferential enrichment is not selectively enriched in the mutant VAPB inclusions. Scale bars: 10 μm (**A**, **H**), 25 μm (**E**).

Being a misfolded ER membrane protein, VAPB-P56S is likely to be degraded by the ERAD-C pathway, i.e. ER associated degradation (ERAD) for ER substrates exposing a misfolded domain into the cytoplasm [47,48]. This pathway like other ERAD pathways involves polyubiquitination of the substrate, followed by extraction from the ER membrane for delivery to the proteasome. Studies in Drosophila and mammalian cells have documented that P56S-VAPB can be polyubiquitinated and that mutant VAPB inclusions are immunostained with antibodies against ubiquitinated epitopes [5,20,49], although in our hands VAPB-P56S inclusions in transfected HeLa cells were relatively weakly positive for ubiquitinated epitopes as compared to inclusion formed by mutant huntingtin [6]. However, using the monoclonal antibody FK2 that immunoreacts with mono- and polyubiquitinated epitopes, mutant VAPB inclusions in our VAPB-P56S mice were strongly stained (Figure 6A). Interestingly, motor neurons without inclusions in our VAPB-P56S mice showed a diffuse increase of FK2-immunostaining throughout the cytoplasm as compared to motor neurons from non-transgenic mice. This was observed in 2 independent experiments with spinal cord sections from both VM1 and VM3 mice and their respective non-transgenic littermates (Figure 6B-D). These data raise the possibility that also diffusely distributed mutant VAPB which is not in the inclusions is ubiquitinated.

We next stained for Valosin-containing protein (VCP/p97, cdc48 in yeast), which is an essential ERAD component that provides mechanical force for extracting substrate from the ER membrane [48]. In wild-type motor neurons VCP-immunoreactivity was present in the nucleus and the perikaryon, with higher staining intensities of the nucleus (Figure 6I). Motor neurons with mutant VAPB inclusions showed the same overall staining, but in addition showed intense VCP staining within the inclusions, indicative of an accumulation of VCP in the inclusions (Figure 6E). Staining for Derlin-1, an ER membrane spanning protein that plays a role in ERAD of many substrates, including tail-anchored proteins [50], showed that VAPB inclusions were also immunoreactive for Derlin-1 (Figure 6F). However, unlike VCP, Derlin-1 immunoreactivity was not specifically enriched in the inclusions, showing the same overall distribution in non-transgenic motor neurons and motor neurons with inclusions (compare Figure 6F’ and H). Finally, we stained for BAP31 an ER membrane chaperone that may play a role in ERAD [24,51], and is enriched in mutant VAPB inclusions in transfected HeLa cells [52]. Accordingly, we found a marked enrichment of BAP31 immunoreactivity in mutant VAPB inclusions in our transgenic mice (Figure 6G). Together the data suggest that mutant VAPB inclusions are a region of increased ERAD activity.

### VAPB inclusions may represent an ER associated degradation (ERAD) quality control compartment

**Figure 7:**
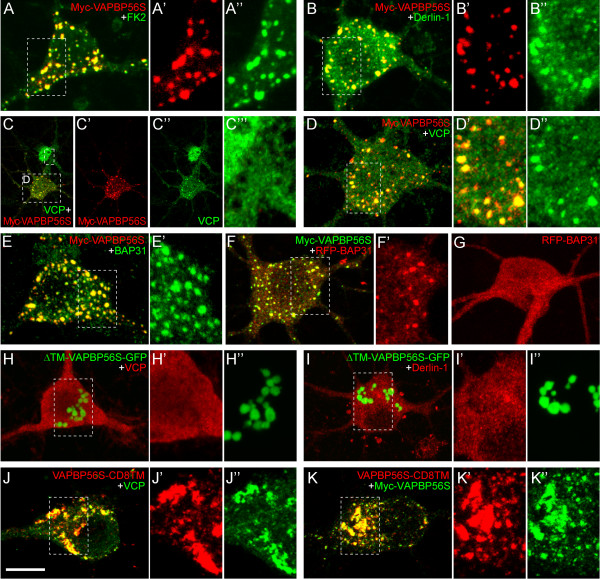
**Mutant VAPB inclusions in primary cultured hippocampal neurons are enriched in ERAD components. A**-**E**) Maximal projections of confocal stacks of primary cultured hippocampal neurons transfected with Myc-VAPB-P56S showing intense staining of ubiquitinated epitopes (FK2, **A**), derlin-1 (**B**), VCP (**C**, **D**) and BAP31 (**E**) in mutant VAPB inclusions. **F**, **G**) Co-expression of Myc-VAPB-P56S and RFP-BAP31 shows that mutant VAPB inclusions recruits a large portion of RFP-BAP31 (**F**), while in single RFP-BAP31 transfected neurons, RFP-BAP31 has a diffuse distribution (**G**). **H**, **I**) Hippocampal neurons transfected with GFP-tagged VAPB-P56S lacking the transmembrane domain (ΔTM) develop inclusions that are immunonegative for VCP (**H**) and Derlin-1 (**I**). **J**, **K**) Transfection of hippocampal neurons with VAPB-P56S-CD8TM with (**K**) or without (**J**) myc-VAPB-P56S (green) shows that VAPB-P56S with the transmembrane domain of CD8 (immunostained with anti-CD8 antibody) accumulates in the same inclusions as VAPB-P56S. Scale bar, 10 μm.

To further study the relationship between the ERAD pathway and the mutant VAPB inclusion we moved to primary neuron cultures. Like mutant VAPB inclusions in transgenic mouse motor neurons, inclusions in hippocampal neurons transfected with myc- or HA-tagged VAPB-P56S were markedly enriched in ubiquitinated epitopes, VCP, Derlin-1 and BAP31 (Figure 7A-E). The recruitment of BAP31 to the mutant VAPB inclusions was confirmed by coexpressing a RFP-tagged BAP31 (Figure 7F, G). To determine whether the recruitment of ERAD factors to mutant VAPB inclusions depended on the insertion of VAPB-P56S in the ER membrane, neurons were transfected with GFP-tagged VAPB-P56S that lacked the transmembrane domain required for insertion in the ER membrane [52]. ΔTM-VAPB-P56S accumulated in inclusions that usually were larger and showed a more restricted distribution than VAPB-P56S inclusions (Figure 7H, I). Importantly, ΔTM-VAPB-P56S inclusions were not enriched in VCP and Derlin-1 (Figure 7H, I) indicating that the recruitment of these ERAD factors depended on the association of mutant VAPB with the ER. In contrast, VAPB-P56S with the transmembrane of another protein (i.e. CD8) accumulated in inclusions that were enriched in VCP (Figure 7J). Furthermore, co-transfection of VAPB-P56S-CD8TM and Myc-VAPB-P56S showed that VAPB-P56S-CD8TM accumulated in the same inclusions as VAPB-P56S (Figure 7K) indicating that the ER-membrane proteins with different transmembrane domains may accumulate in the same ERAD-associated inclusions.

**Figure 8:**
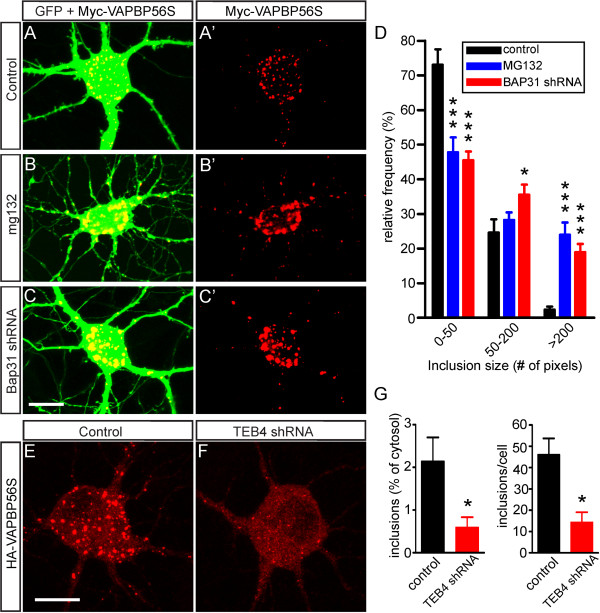
**Altering ERAD influences the size of mutant VAPB inclusions. A**-**D**) Incubation of GFP and Myc-VAPB-P56S transfected primary cultured hippocampal neurons with MG132 to inhibit proteasome (**B**), or BAP31 shRNA construct to down regulate BAP31 expression (**C**) result in increased size of mutant VAPB inclusions as compared to untreated cultures (**A**). The relative frequencies of small (< 50 pixels) and large (>200 pixels) were reduced and increased, respectively, by MG132 and BAP31 shRNA treatment (**D**). * and ***, P < 0.05 and <0.0001 compared to control; one-way ANOVA, Tukey’s post-test. **E**-**G**) TEB4 shRNA reduces size and number of mutant VAPB inclusions in cultured hippocampal neurons, also resulting in a reduction of the proportion of cytosol occupied by inclusions in TEB4-shRNA treated cells (**G**). *, P < 0.05 compared to control (Student’s *t*-test). Scale bar, 10 μm.

Inhibition of the ERAD pathway by inhibiting the proteasome with MG-132 resulted in the formation of larger inclusions (Figure 8A-D). Also shRNA-mediated knockdown of BAP31 resulted in larger inclusions (Figure 8C). Next we studied the effect of knockdown of TEB4 (MARCH-VI), an ER membrane bound E3 ubiquitin ligase [53] that is the mammalian ortholog of yeast Doa10, which is required for ubiquitination of ERAD substrates with defective cytosolic domains [54]. ShRNA-mediated TEB4 knockdown resulted in a dramatic reduction in the size and number of mutant VAPB inclusions in VAPB-P56S expressing neurons (Figure 8E-G). Together these data indicate that the sizes of the VAPB-P56S inclusions strongly depend on ERAD activity.

### VAPB-P56S inclusions differ from an ER protective organelle (ERPO) associated with luminal ERAD substrates

**Figure 9:**
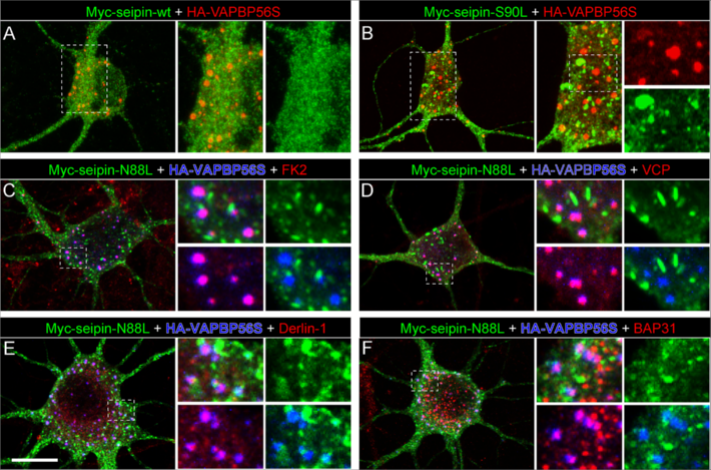
**Mutant VAPB inclusions do not colocalize with mutant seipin inclusions.** Primary cultured hippocampal neurons cotransfected with HA-VAPB-P56S and wild-type (**A**) S90L- (**B**), or N88L-mutant myc-tagged seipin constructs (**C**-**F**). Wild-type seipin shows a diffuse distribution throughout the cell body (**A**), while seipin-S90L and seipin-N88S accumulate in small inclusions (**B-E**) resembling mutant VAPB inclusions. However, seipin inclusions do not overlap with VAPB inclusions, and do not stain with antibody against ubiquitinated epitopes (FK2, **C**), VCP (**D**), derlin-1 (**E**) and BAP31 (**F**). Scale bar, 10 μm.

The data indicate that VAPB-P56S inclusions in motor neurons of our P56S-VAPB transgenic mice represent a defensive cell response aimed at protecting cells from a level of mutant ER protein that exceeds the capacity of ER associated degradation. A recent study documented a protective ER compartment termed ERPO (ER protective organelle) in neurons of mice expressing mutated forms of the ER membrane protein seipin that are associated with an autosomal dominant motor neuron disease termed seipinopathy [27,55]. The two seipinopathy mutations, N88S and S90L, are located within an ER luminal loop of the protein, disrupt a glycosylation site and facilitate aggregation. Consistent with previous reports expression of seipin-S90L and seipin-N88S in cultured neurons resulted in small spherical inclusions with sizes in the same range as VAPB inclusions. However, coexpression of seipin-N88S or seipin-S90L with VAPB-P56S showed that the mutated proteins accumulated in distinct inclusions (Figure 9). The seipin inclusions were not immunostained with the FK2 antibody against ubiquitinated epitopes. Furthermore, the seipin inclusions unlike the VAPB inclusions were immunonegative for VCP, derlin-1 and BAP31. These data show that VAPB inclusions are different from ERPO formed by mutant seipin.

## Discussion

### Inclusions in motor neurons of transgenic VAPB-P56S mice may represent an ER quality control compartment

In the present study we show that transgenic mice expressing an ALS8-linked mutant form of VAPB develop a novel type of inclusion that is associated with the rough ER and consists of smooth ER-like tubular profiles and electron dense material. Several lines of evidence indicate that this mutant VAPB inclusion represents an ER protein quality control compartment: First, the presence of the inclusions was not associated with signs of neuronal malfunction or neuronal pathology. Second, the inclusions are localized in the center of healthy appearing active rough ER. Third, the inclusions are reversible as they gradually disappear following axonal transection. Fourth, the inclusions are enriched in factors that operate in the ER associated degradation (ERAD) pathway, i.e. p97/VCP, Derlin-1 and the ER membrane chaperone BAP31. And fifth, inhibition of ERAD increased the size of the inclusions. We propose that the inclusions in our mutant VAPB transgenic mice represent an ER quality control compartment that arises, when the amount of substrate exceeds the capacity of ER associated degradation (ERAD). This ERAD associated quality control compartment is reminiscent of aggresomes that may form in condition of excess of cytosolic aggregation-prone protein [4,56]. Compatible with this idea, mutant VAPB inclusions do not occur in motor neurons of low-expressing mutant VAPB transgenic mice (line VM2), and are absent in motor neurons derived from induced pluripotent stem cells of ALS8 patients [57].

Several studies have reported on ER-derived inclusion-like structures that are formed after expression of mutated ER proteins in yeast or mammalian cells [55,58-60]. An ER-derived structure termed ERPO (ER protective organelle) has been identified as a protective ER compartment in cells expressing the serpin α1-antitrypsin with an E342K mutation, associated with liver disease in children [58]. ERPO may represent an ER quality control pathway for multiple ER substrates, since aggregation-prone mutant forms of seipin that are associated with an autosomal dominant motor neuron disease, accumulate in the same compartment [27]. Our data show that mutant VAPB and mutant seipin accumulate in different inclusions when coexpressed, indicating that the mechanisms that operate in the formation of VAPB inclusions differ from those underlying the formation of ERPO. Other ER derived degradation subcompartments include a quality control compartment for misfolded glycoproteins, termed ERQC [60] and a compartment termed ERAC (ER associated compartment) that is formed in yeast expressing a multispanning membrane protein (Ste6p) with mutation in cytoplasmatic domains [59]. ERAC comprises a network of tubulo-vesicular structures that occasionally are continuous with the ER profiles, and was found to prevent proteins targeted for ERAD from entering the secretory pathway [59]. These properties are compatible with features of VAPB inclusions. A hallmark of our VAPB inclusions is their position in the center of normal appearing RER that in motor neurons usually is organized in Nissl Bodies [61] (Figure 2). This position indicates that mutant VAPB is sorted in a direction opposite to the secretory pathway. Further work is needed to determine whether mutant VAPB inclusions represent an ERAC-like compartment, and whether there are additional ER substrates with misfolded cytosolic domains that accumulate in the same structures. Our finding that mutant VAPB with a different ER transmembrane domain accumulate in the same structures (Figure 7J, K) favors the notion that the mutant VAPB inclusions represent a protective ‘waste basket’ for multiple ERAD-C substrates.

The molecular mechanisms underlying the formation of mutant VAPB inclusions remain to be further determined. Previously, we have shown that their formation does not require microtubule-dependent transport, which is instrumental for the formation of aggresomes and several other quality control compartments [4,6]. Here, we show that shRNA-mediated knock-down of the ER membrane bound E3 ligase TEB4 (MARCH-VI) severely reduces the size and number of mutant VAPB inclusions. TEB4 and its yeast ortholog Doa10, mediate ubiquitination of multiple ERAD-C substrates [53,54], and accordingly TEB4 may mediate ubiquitination of mutant VAPB to target it for the ERAD machinery [54,62-64]. These data suggest that ubiquitination or another activity of TEB4 is an early step in the formation of VAPB inclusions. We also found that knock-down of the ER membrane chaperone BAP31 increases the size of VAPB inclusions. BAP31 has been implicated in ERAD [24,51], accumulates in the mutant VAPB inclusions, and may be involved in the interaction of ubiquitinated mutant VAPB with VCP. In this scenario the absence of BAP31 would prevent efficient extraction of mutant VAPB from the ER membrane by VCP. Some proteins that interact with the transmembrane domain of mutant VAPB, e.g. wild-type VAPs [6] and YIF1A [65], are recruited to the VAPB inclusions, raising the question what happens with these proteins during ERAD of mutant VAPB. Another question is how the formation of ER quality control compartments such as VAPB inclusions, are connected to unfolded protein response pathways that may be activated in conditions of proteotoxic ER stress and overload of ERAD, and that have been implicated in ALS pathogenesis [66].

### Wild-type VAPB overexpression causes stacked ER

Recent work of Borgese and co-workers [5,52] suggests that VAPB-P56S inclusions in HeLa cells predominantly consist of a special form of stacked ER, made of two or three tightly apposed ER cisternae separated by an electron-dense layer [5,52]. We did not observe this or any other form of stacked ER in our mutant VAPB expressing lines. VAPB inclusions in HeLa cells are also BAP31 [52] and VCP/p97-positive (data not shown), and mutant VAPB was shown to be rapidly ubiquitinated, and degraded in a proteasome and VCP/p97 dependent way [5], suggesting that as in neurons mutant VAPB inclusions may represent an ERAD-associated compartment. Renewed ultrastructural analysis of our HeLa cells showed that in cells with relatively few and small inclusions they resembled the inclusions observed in mutant VAPB mice (Additional file 1: Figure S6A, B), while in cells with more and larger inclusions they showed more complex morphologies (Additional file 1: Figure S6C-E). Interestingly, we noted patches of apposed ER cisternae separated by a thin layer of electron dense cytosol, resembling the apposed ER cisternae reported by Borgese and coworkers. However, so far in our HeLa cells we have not identified the relatively large domains of bi- or trilaminar ER documented by Borgese et al. [5,52]. Hence, the precise relationship between our VAPB inclusions and the remodeled stacked ER of Borgese et al. remains to be determined.

Stacked ER occurred in motor neurons of our wild-type hVAPB transgenic mice, and is a well-documented phenomenon in cells that coexpress wild-type VAPB and FFAT-motif proteins, presumably resulting from heterotypic interaction between VAPB and FFAT-motif proteins [41,42]. Hence, stacked ER in motor neurons of wild-type VAPB overexpressing mice may result from excessive VAPB interacting with endogenous FFAT-motif protein. Remarkably, in one line of wild-type VAPB overexpressing mice we observed a variant of stacked ER where the cytosolic space linking the cisterns was considerably more electron dense. These data indicate that stacked ER in some conditions is irreversible, which contrasts with the notion that stacked ER is a relatively harmless and reversible phenomenon [39,40].

### How does mutant VAPB cause motor neuron disease?

Consistent with previous studies [17,18] our data show that neuron-specific mutant VAPB transgenic mice generally do not develop motor symptoms and signs of motor neuron degeneration. This contrasts with the pathological phenotypes observed in drosophila expressing mutant VAPB (DVAP-P58S or DVAP-T48I) that develop loss of function-like phenotypes, and are suggestive of a dominant-negative mode of action of mutant VAPB [6,7,19,49,67]. The absence of a phenotype in mice may be explained by efficient degradation and the accumulation of mutant VAPB in a protective compartment that prevents mutant VAPB from accumulating at sites where it could engage in aberrant interactions such as the ER Golgi intermediate compartment (ERGIC), or the axon. The same mechanisms may explain the late and variable onset of disease in man [8,68,69]. Interestingly, two (out of 46) mice of our aging cohort developed a late onset motor axonopathy that is reminiscent of mutant VAPB induced disease in man. Although their number is too low to draw conclusions, a striking feature of these mice with motor axon pathology was the absence of VAPB inclusions, indicative of a negative correlation between the presence of inclusions and the development of pathology. However, in view of our finding that inclusions gradually disappear in axotomized motor neurons, an alternative explanation is that the inclusions in the mice with motor axon pathology have disappeared secondarily to the axonal pathology. Further work is needed to determine the role of protective pathways like ERAD and the formation of inclusions in preventing disease onset in mutant VAPB expressing mice, as well as the factors that cause the disappearance of VAPB inclusions after axotomy.

A recent study with *Vapb*^*−/−*^ mice has indicated that VAPB deficiency leads to mild, late onset defects in motor performance, but does not cause neuromuscular junction abnormalities and muscle denervation [9]. These data suggest that loss of VAPB function by itself is not sufficient to trigger an ALS-like disorder perhaps because of compensatory actions by VAPA [9] and point to alternative or additional mechanisms for mutant VAPB toxicity, such as a gained toxic activity, or a dominant-negative effect. It would be interesting to cross VAPB-P56S transgenic mice with *Vapb*^*−/−*^ mice to examine the presence of synergistic deleterious interactions between the absence of VAPB and the presence of mutant VAPB.

## Conclusions

In conclusion, the central finding of the present study is that inclusions formed by ALS8-mutant VAPB in motor neurons in transgenic mice represent a protective ER compartment that isolates misfolded and aggregated VAPB from the rest of the ER. The data suggest that motor neurons are capable of coping with mutant VAPB levels that exceed the capacity of the ERAD systems. Whether similar protective ER derived compartments occur in physiological and pathological conditions in human central nervous system could be analyzed by immunohistological approaches with antibodies against BAP31 and VCP/p97.

## Competing interests

The authors declare that they have no competing interests.

## Authors’ contributions

MK, VvD, CCH and DJ designed research; MK, VvD and EDH performed experiments with transgenic mice; MK performed experiments with cultured neurons; MH, KV and JAP performed electron microscopy on HeLa cells; WS performed unfolded protein response analyses; MK, VvD and DJ analyzed the data; MK, VvD and DJ wrote the paper; CCH and DJ supervised the project. All authors read and approved the final manuscript.

## Supplementary Material

Additional file 1 Supplementary Data of ‘Amyotrophic lateral sclerosis (ALS)-associated VAPB-P56S inclusions represent an ER quality control compartment’. Click here for file
