# Supplementary material for: Amyotrophic lateral sclerosis (ALS)-associated VAPB-P56S inclusions represent an ER quality control compartment
Source: Acta Neuropathol Commun. 2013 Jun 12;1:24. doi: 10.1186/2051-5960-1-24 (PMC3893532; doi:10.1186/2051-5960-1-24)
Supplement: Additional file 1 — Supplementary Data of ‘Amyotrophic lateral sclerosis (ALS)-associated VAPB-P56S inclusions represent an ER quality control compartment’. [file 2051-5960-1-24-S1.pdf]

Supplementary Data of 'Amyotrophic lateral sclerosis (ALS)-associated VAPB-P56S inclusions represent an ER quality control compartment'

Marijn Kuijpers, Vera van Dis, Elize D. Haasdijk, Martin Harterink, Karin Vocking, Jan A. Post, Wiep Scheper, Casper C. Hoogenraad, Dick Jaarsma

Number of Supplementary Tables: 1

Number of Supplementary Figures: 6

Table S1: Cohort of mutant VAPB transgenic mice tested for the development of motor abnormalities up to 104 weeks of age

Mouse line	mice included (male/female)	mice killed because of non-motor discomfort (%)	mice in the study	mice developing late onset motor deficits (%)
**non-tg**	23 (11/12)	4 (17%)	19	0
**hVAPB-P56S**				
**VM1**	12 (8/4)	1 (8%)	11	0
**VM2**	9 (5/4)	2 (22%)	7	0
**VM3**	9 (4/5)	0	9	1 (11%)
**VM5**	11 (8/3)	2 (18%)	9	0
**VM1+VM5**	11 (6/5)	1 (9%)	10	1 (10%)
**total**	52 (31/21)	6 (12%)	46	2 (4%)

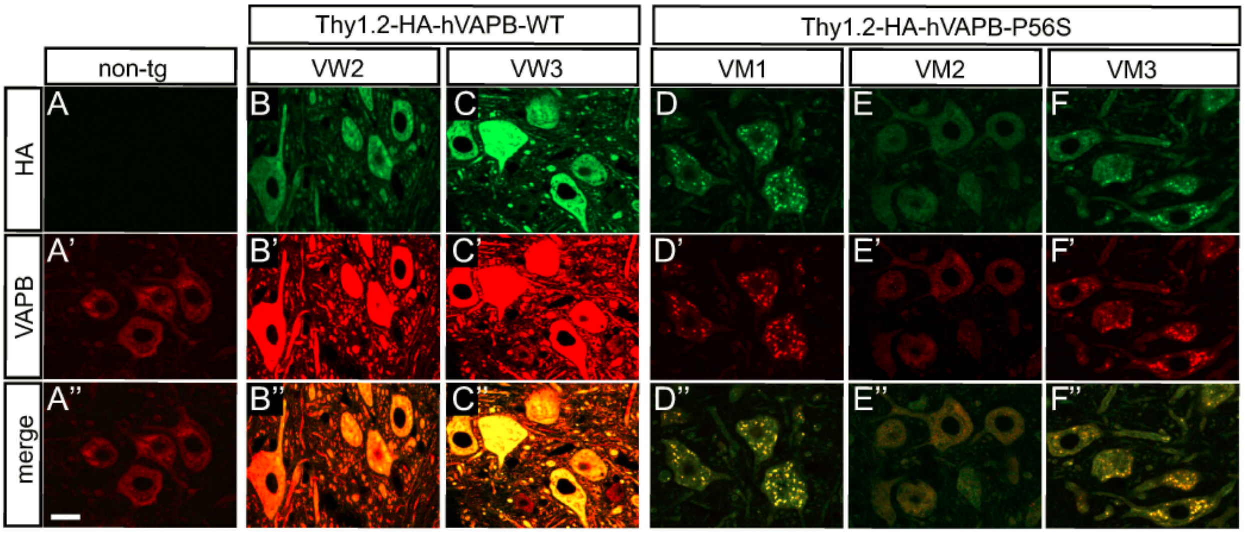

Figure S1. VAPB-P56S transgenic mice develop inclusions in motor neurons

Confocal double-labeling immunofluorescence of HA and VAPB in lumbar L4 sections of wild-type (VW2, VW3) and mutant (VM1, VM2, VM3) VAPB transgenic mice showing high and moderate transgene expression in L4 spinal motor neurons of wild-type (B, C) and mutant (D-F) transgenic mice, respectively. Note multiple small spherical intensely HA and VAPB-immunoreactive inclusions in VM1 (D) and VM3 (F) motor neurons and the absence of these structures in VM2 motor neurons (E). Bar in A", 10 µm.

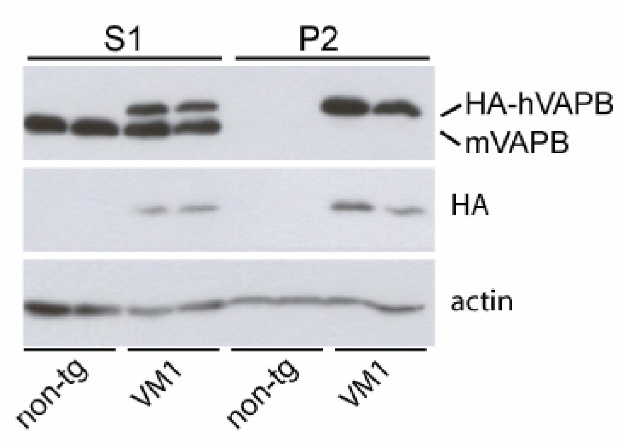

Figure S2. Altered solubility of VAPB-P56S in VAPB-P56S transgenic mice

Western blot of whole homogenates (S1), and Nonidet P40-insoluble fraction (P2) of spinal cord from mutant VAPB (line VM1) and non-transgenic mice showing the accumulation of mutant VAPB (but not endogenous mouse VAPB) in the P2 fraction. Endogenous murine VAPB was not detectable in this fraction, suggesting that it does not coaggregate with transgenic mutant VAPB.

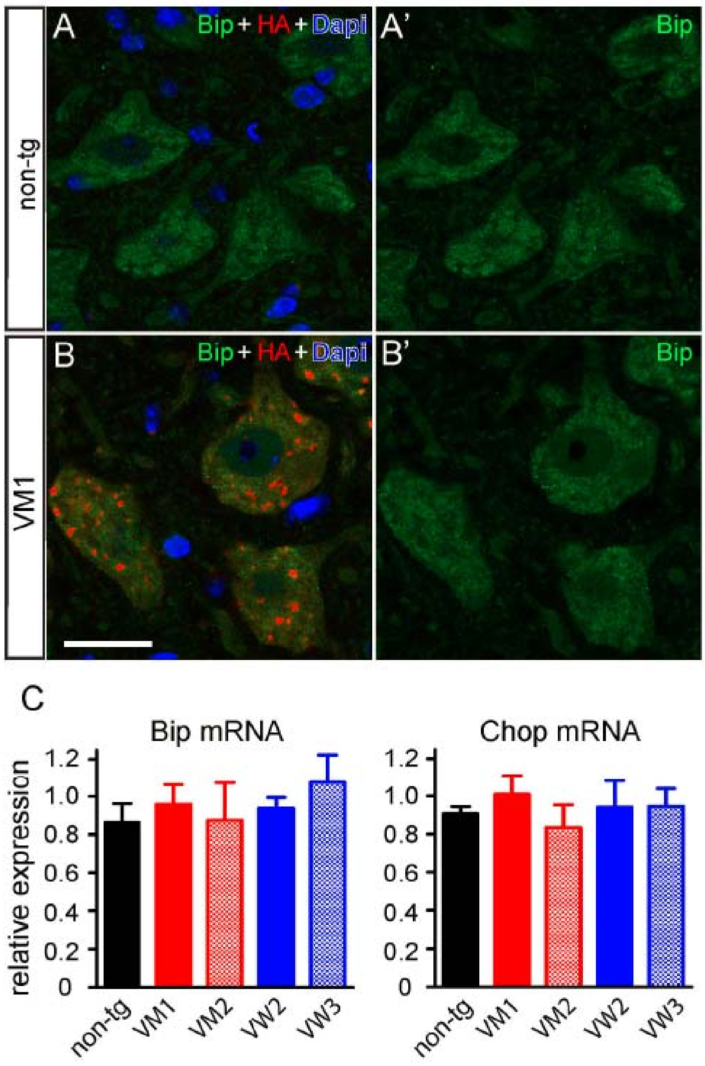

Figure S3. VAPB-P56S overexpression does not affect the expression of unfolded protein responsive genes

A, B) Confocal double-labeling immunofluorescence of HA and VAPB in lumbar L4 sections of non-transgenic and mutant VAPB transgenic mice (line VM1, B) showing unaltered expression of the ER chaperone Bip (Grp78, Hspa5) in motor neurons with VAPB inclusions. Scale bar in B, 20 µm.

C) Bar graphs showing expression levels of BiP and Chop mRNA in neocortex of wild-type (VW2, VW3) and mutant (VM2, VM2) VAPB transgenic mice determined using quantitative RT-PCR. Values are means ± SE from 3-4 mice, and are normalized to eEF2α mRNA for individual animals.

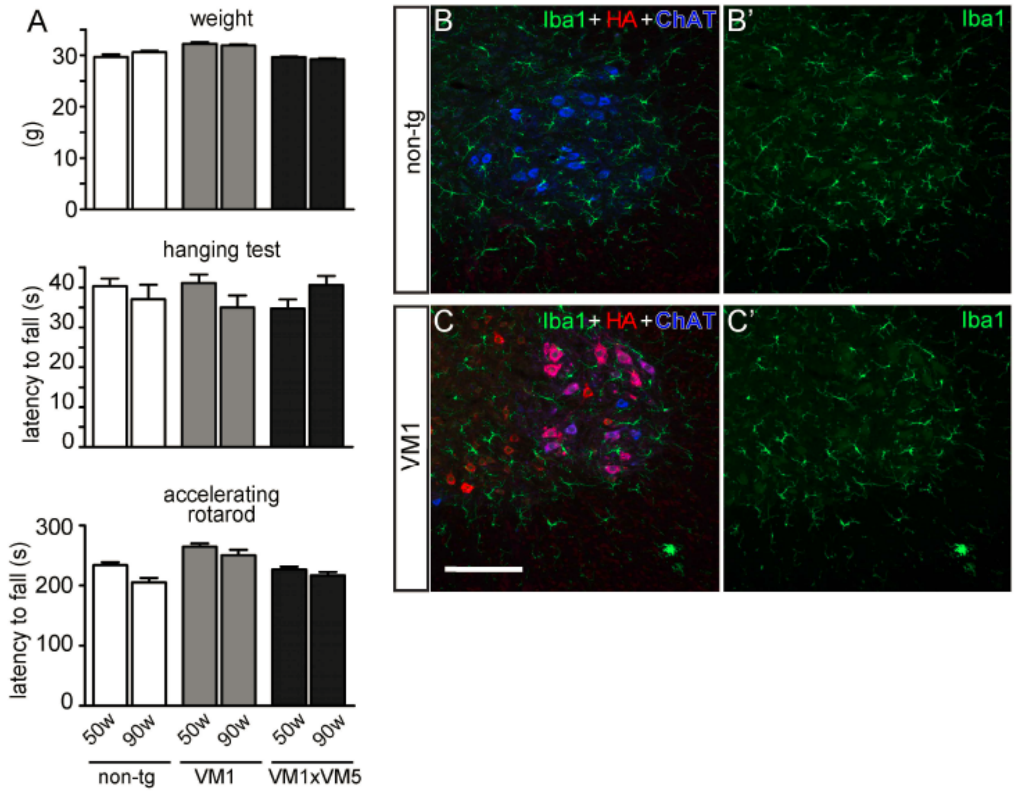

Figure S4. Absence of motor abnormalities and signs of neuronal degeneration in most VAPB-P56S transgenic mice

A) Bar graphs showing weight and motor performance in hanging grid and accelerating rotarod tests of mutant VAPB transgenic mice (line VM1 and VM1xVM5 mice) and non-transgenic littermates. VAPB transgenic mice showed the same weight and motor performance as non-transgenic littermates. Values represent means ± SE (n = 11, 10 and 8 for non-tg, VM1 and VM1 x VM5 mice, respectively).

B, C) Confocal triple-labeling immunofluorescence of HA, Iba1 (microglia cells), and ChAT (motor neurons) showing unaltered expression of Iba1 in 104 weeks old VM1 mice as compared to non-transgenic littermates, which is indicative for the absence of microgliosis in mutant VAPB transgenic mice. Scale bar, 100 µm.

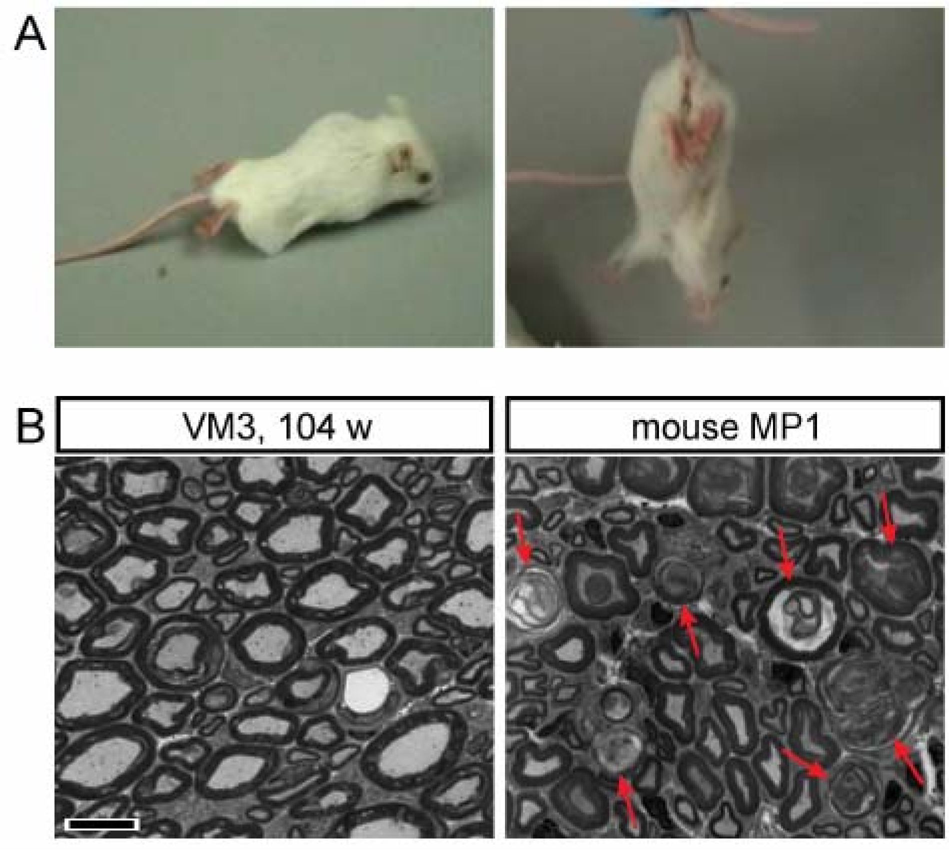

Figure S5. Late onset progressive motor abnormalities and axonal degeneration in 2 VAPB-P56S transgenic mice

A) Of a cohort of 46 VAPB-P56S transgenic mice derived from different lines (VM1, VM2, VM3, VM5 VM1xVM5) 44 reached the age of 2 years without developing signs of motor deficits as measured by accelerating rotarod, hanging wire and hind limb extension tests, while 2 mice developed progressive motor impairment. The mice with a motor phenotype (MP) included a mouse from line VM3 (mouse MP1, onset 61 weeks) and line VM1xVM5 (mouse MP2, onset 74 weeks). The mice were killed when they were unable to hang for more than 1s and started to show > 20% weight loss at the age of 65 (mouse MP1) and 78 (mouse MP2) weeks. Panel A shows mouse MP2 with abnormal positioned hind limbs during locomotion and when lifted by their tails.

B) Semithin (0.5 µm) toluidine blue-stained sciatic nerve sections of mutant VAPB mice (line VM3) showing degenerating axons (red arrows) in mouse MP, a VM3 mouse that developed muscle weakness.

Scale bar: 2 µm.

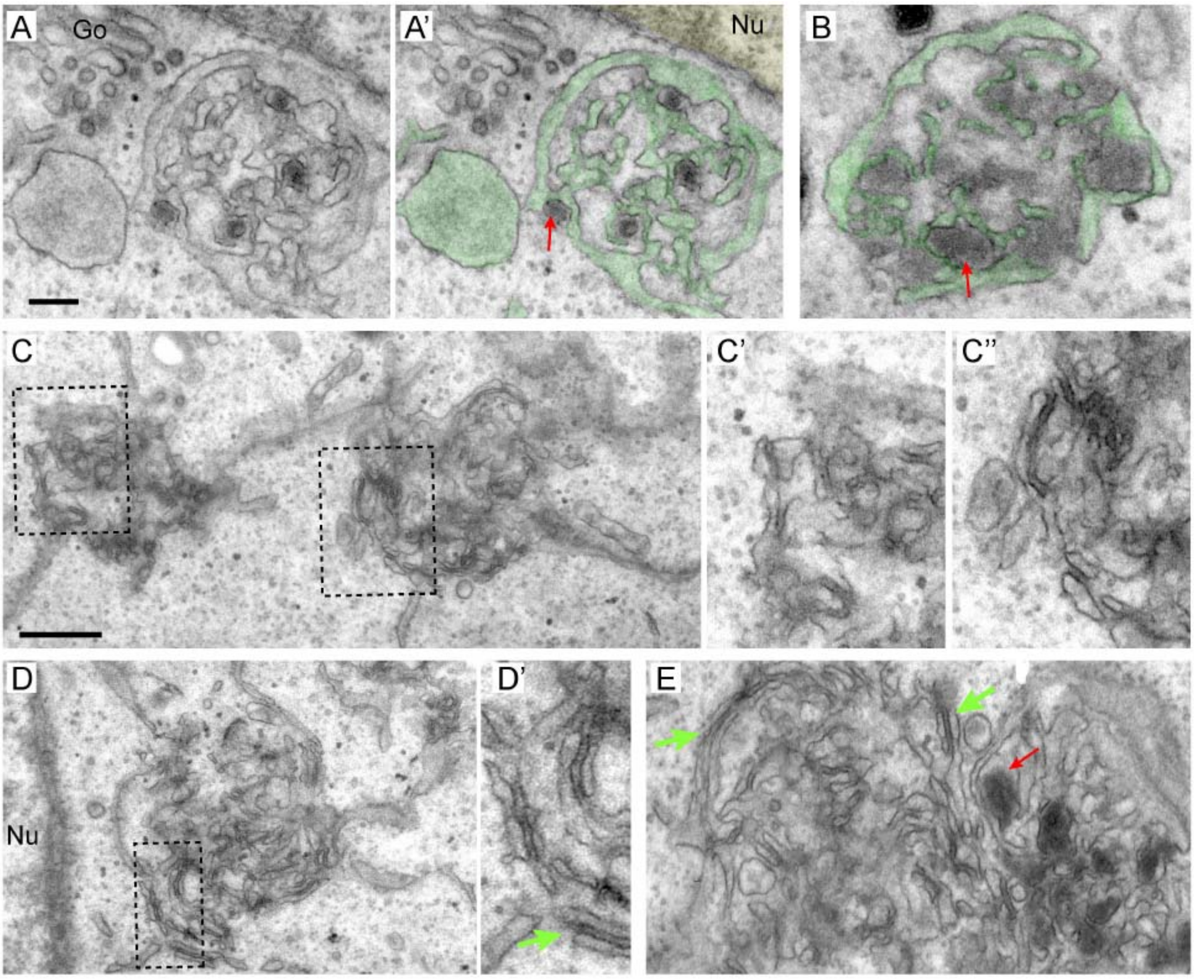

Figure S6. Mutant VAPB inclusions in HeLa cells consist of smooth ER-like membranous profiles and electron dense material

Transmission electron photomicrographs of mutant VAPB inclusions in HeLa cells 24 h after transfection with Myc-VAPB-P56S. The structures shown in A and B were identified in cells with relatively few inclusions (1-3 per cell cross section) and consist of an ensemble of tubular and vesicular ER profiles that in many occasions are continuous with surrounding ER. Typically the cytosol between these tubular profiles contained patches of electron dense material (small red arrows). The structures shown in C-E were identified in cells with a higher density of inclusions (4-15 per cell cross section) with larger diameters. These inclusions typically showed higher packing density of membranous profiles, that in many occasions were organized in small stretches of apposed ER cisternae separated by a thin layer of electron dense cytosol (green arrows in D' and E).

Color overlay in A' and B: yellow, nucleus (Nu); green, lumen of ER.

Scale bars: 250 nm (A), 500 nm (C).
